# Galectin-3, transforming growth factor beta 1, and brain natriuretic peptide in cardiac remodeling under hyperlipidemic and hyperglycemic stress

**DOI:** 10.1007/s11010-026-05620-z

**Published:** 2026-07-01

**Authors:** Hina Patel, Angelie Pathak, Resmi Rajalekshmi, Brian David, Devendra K. Agrawal

**Affiliations:** Department of Translational Research, College of Osteopathic Medicine of the Pacific, Western University of Health Sciences, 309 E. Second Street, Pomona, CA 91766 USA

**Keywords:** Brain natriuretic peptide, Cardiac remodeling, Cardiomyopathy, Fibrosis, Galectin-3/TGF-β1 signaling, Hyperglycemia, Hyperlipidemia, Metabolic cardiomyopathy, Pathologic cardiac hypertrophy

## Abstract

Hyperlipidemia (HYL) and hyperglycemia (HYG) directly drive pathological cardiac remodeling; however, the mechanisms by which these conditions differentially regulate profibrotic signaling and early stress‑response pathways in left ventricular (LV) myocardium remain poorly defined. This study investigated the expression and localization of galectin-3 (Gal-3), transforming growth factor-β1 (TGF-β1), and brain natriuretic peptide (BNP) in a Yucatan miniswine model of HYL and HYG. Female Yucatan miniswine were assigned to normal control, HYL (high-cholesterol diet), or HYG (high-fat/high-carbohydrate diet plus low dose streptozotocin) groups (*n* = 6/group) for 8-weeks, and LV tissue was assessed by histology, qRT-PCR, Western blot, and immunohistochemistry. Both metabolic conditions induced cardiomyocyte hypertrophy and interstitial fibrosis, with more diffuse collagen deposition in HYG myocardium. qRT-PCR demonstrated upregulation of Gal-3, TGF-β1, and BNP relative to controls, with transcript levels positively correlating with circulating glucose concentration. Western blot analysis showed relatively greater active TGF-β1 signal in HYL myocardium and greater latent precursor accumulation in HYG myocardium, suggesting condition-associated differences in TGF-β1 regulation. Immunohistochemistry demonstrated perivascular Gal-3 localization, cytoplasmic and perinuclear TGF-β1 immunoreactivity in cardiomyocytes, and BNP immunoreactivity predominantly within subendocardial Purkinje fibers, with increased staining intensity in metabolically stressed groups compared with controls. Collectively, these findings suggest that HYL and HYG are associated with overlapping but distinct patterns of Gal-3, TGF-β1, and BNP expression during metabolic cardiac remodeling. The Gal-3/TGF-β1 axis may represent a shared profibrotic pathway, whereas BNP may reflect a compensatory stress response whose functional significance requires further mechanistic validation.

## Introduction

Cardiac remodeling is defined as the structural and functional alterations of the myocardium that occur in response to sustained hemodynamic, metabolic, or inflammatory stress. Adult cardiomyocytes possess a limited regenerative capacity in response to increased workload and therefore preserve cardiac output through hypertrophic growth [1–3]. Hypertrophy is characterized by an increased size of individual cardiomyocytes. This represents a compensatory mechanism that allows the heart to maintain contractile function under increased physiological demand [4–7]. Physiologic conditions like postnatal growth, nutrition, exercise, vitamin D deficiency, and pregnancy can influence hypertrophic remodeling, which is typically reversible and associated with preserved cardiac function [8–11]. However, hypertrophic remodeling becomes maladaptive under persistent pathological stress and is accompanied by excessive extracellular matrix (ECM) deposition, fibrosis, increased myocardial stiffness, poor cardiac performance, and ultimately, heart failure [2, 6, 12–16].

Fibrosis is a central feature of pathological cardiac remodeling and is not seen in physiologic stress [17–20]. The ECM in a healthy heart consists of a highly organized fibrillar collagen network within the myocardium [21–23]. This network is composed of collagen types I and III that provide structural support and maintain proper myocardial alignment during both diastole and systole [24]. In contrast, under pathological conditions, fibroblasts are activated and differentiate into myofibroblasts. This leads to excessive collagen deposition and remodeling of the myocardial interstitium. Fibrosis disrupts normal myocardial architecture, impairs oxygen diffusion, and increases myocardial stiffness, placing a greater mechanical stress on cardiomyocytes [25, 26]. This establishes a cycle of cardiomyocyte dysfunction, hypertrophy, and progressive fibrotic remodeling that may be worsened by pathological conditions like systemic metabolic disorders [18].

Hyperlipidemia (HYL) and hyperglycemia (HYG) are well-established metabolic risk factors for cardiovascular disease and heart failure. Hyperlipidemia contributes to cardiac remodeling through lipotoxicity and metabolic derangements [27, 28]. Elevated free fatty acid levels promote lipid accumulation within myocardial and vascular tissues, generating toxic metabolic intermediates, macrophage infiltration, oxidative stress, and chronic inflammation [29, 30]. Similarly, hyperglycemia induces the formation of advanced glycation end products (AGEs), mitochondrial dysfunction, and reactive oxidative species, which activate inflammatory and profibrotic signaling pathways [31, 32].

Collectively, these metabolic disturbances disrupt normal myocardial homeostasis and contribute to oxidative damage, endothelial dysfunction, inflammation, and adverse cardiac remodeling. These processes are mediated by complex molecular signaling networks that regulate myocardial stress responses. Galectin-3 (Gal-3), transforming growth factor beta 1 (TGF-β1), and brain natriuretic peptide (BNP) are among the key mediators involved in these pathways. Gal-3, a β-galactoside-binding lectin secreted primarily by activated macrophages, is an important regulator of fibroblast activation and extracellular matrix deposition [33]. Gal-3 is closely associated with TGF-β1, a central profibrotic cytokine that drives ECM remodeling by promoting myofibroblast differentiation and collagen synthesis. Prior experimental studies have shown that Gal-3 can activate the TGF-β1/Smad signaling pathway, amplifying fibrotic responses [34, 35]. In contrast, BNP is released mainly by ventricular cardiomyocytes in response to myocardial stretch and increased wall stress. BNP functions as a compensatory hormone with natriuretic, vasodilatory, and antifibrotic effects that may help limit pathological remodeling [36, 37].

Although these molecular mediators have been independently involved in cardiac remodeling, the mechanisms by which upstream metabolic stressors such as HYL and HYG influence their expression and interaction within the myocardium remain incompletely understood. While previous studies have investigated Gal-3-mediated remodeling post-myocardial infarction in a rodent model, little is known about how these pathways respond to chronic metabolic stress in large animal models. Swine models provide valuable insights into human cardiovascular disease because the porcine cardiovascular system closely resembles that of human anatomy, physiology, and hemodynamics. Therefore, this study aimed to investigate the effects of hyperlipidemic and hyperglycemic conditions on cardiac remodeling in a Yucatan miniswine model by evaluating structural changes in the left ventricular myocardium and assessing the expression of Gal-3, TGF-β1, and BNP.

## Materials and methods

### Animal model and left ventricle tissue collection

Experimental procedures were approved by the Institutional Animal Care and Use Committee (IACUC) (Protocol No. R22IACUC034) at Western University of Health Sciences (Pomona, CA, USA). Five-month-old female Yucatan miniswine (Sus scrofa; Premier Bioresources, Ramona, CA, USA; ~30 kg) were acclimatized for two weeks under controlled conditions (72–74 °F; 12 h light/dark cycle) with ad libitum water and twice-daily feedings.

Animals were assigned to three groups (*n* = 6 per group): (i) normal control (standard maintenance diet); (ii) hyperlipidemic (HYL; high-cholesterol [HC] diet: 51% carbohydrate, 4% cholesterol, 20% protein, 10% fat); and (iii) hyperglycemic (HYG; high-fat high-carbohydrate [HFHC] diet: 45% fructose, 5% protein, 45% fat), with two intravenous injections of streptozotocin (STZ; 50 mg/kg per dose via ear vein, one week apart) to induce a type 2 diabetes mellitus phenotype. Fasting blood cholesterol (HYL: 450–700 mg/dL) and glucose (HYG: 100–300 mg/dL) were confirmed at endpoint.

After eight weeks, animals were euthanized under general anesthesia (acepromazine 1.1 mg/kg IM; telazol 2.5–5 mg/kg + xylazine 1–2 mg/kg IM; isoflurane 1–3% maintenance) by intravenous pentobarbital sodium (Euthasol^®^; 1 mL/10 lb). Left ventricular (LV) tissue was harvested and allocated to 10% neutral-buffered formalin (histology), RNAlater^®^ (RNA) (AM7021, ThermoFisher Scientific, Waltham, Massachusetts, USA), and −80 °C (protein).

### Histology processing and staining

Formalin-fixed LV tissue was paraffin-embedded and sectioned at 5 μm (Leica RM2265 microtome). Hematoxylin and eosin (H&E) and Masson’s trichrome staining were performed on deparaffinized, rehydrated sections per standard protocols to assess myocardial morphology and interstitial collagen deposition, respectively. Myocardial fiber diameters were measured for normal, hyperlipidemic, and hyperglycemic swine. Five measurements were taken in a clockwise manner with consistent distribution across all images using Fiji ImageJ.

Stained sections were coverslipped with Cytoseal™ 60 (Thermo Fisher Scientific, Waltham, MA, USA) and imaged at 10× and 20× magnification (Leica DM6, Wetzlar, Germany). For each sample, three consecutive tissue sections were evaluated, and 3–5 representative fields were scanned per section. All images and measurements were blindly reviewed by an independent observer, and quantitative analysis was performed using Fiji ImageJ.

### Quantitative real-time polymerase chain reaction (qRT-PCR)

Total RNA was extracted from ~ 50 mg LV tissue using TRIzol^®^ (catalog no. T9424; Millipore Sigma) per the manufacturer’s protocol. RNA purity (A260/A280 ≥ 1.8) and concentration were assessed by NanoDrop 2000 (ThermoFisher Scientific). cDNA was synthesized from 2 µg RNA using the AzuraQuant™ cDNA Synthesis Kit (catalog no. AZ-1996; Azura Genomics Inc.) and diluted 1:20 before use.

The qRT-PCR was performed using AzuraView™ GreenFast qPCR Blue Mix LR (catalog no. AZ-2350; Azura Genomics Inc.) on a CFX96 Real-Time System (Bio-Rad Laboratories) with the following cycling conditions: 95 °C × 3 min; 40 cycles of 95 °C × 10 s/60 °C × 30 s; melt curve analysis to confirm specificity. The primers for genes of interest and the housekeeping (Table 1) were purchased from Integrated DNA Technologies (Coralville, IA, USA). All reactions were performed in technical triplicate. Relative expression was calculated by the 2^−ΔΔCT^ method, normalized to 18S rRNA, and expressed as fold-change relative to controls.

**Table 1:** Sequences of forward and reverse oligonucleotides used for phenotypic characterization by RT-qPCR

Gene	Forward	Reverse
TGF-β1	5′-GGGCTGGAAGTGGATTCAT-3′	5′ -CTTGCTGTACTGAGTGTCTAGG-3′
BNP	5′-GAATACGCAGCCCCAAGACGAT-3′	5′-ACCTCCTGAGCACATTGCAGC-3′
Gal-3	5′-TGGGGAAGGGAAGAGAGACAGA-3′	5′-GCATCATTGACCGCAACCTTGA-3′
18S rRNA	5′-CCCACGGAATCGAGAAAGAG-3′	5′-TTGACGGAAGGGCACCA-3′

The 18S gene was used as a housekeeping gene to normalize results

### Western blot analysis

Total protein was extracted from 100 mg of LV tissue in RIPA buffer (catalog no. PI89901; ThermoFisher Scientific) supplemented with protease inhibitors (catalog no. A32953; ThermoFisher Scientific), clarified at 14,000 × g (4 °C, 10 min), and quantified by Bradford assay (Bio-Rad Protein Assay Kit II). 15 µg of protein was resolved by 4–15% SDS-PAGE, transferred to PVDF membranes, and blocked in 5% skim milk/TBST (1 h). Membranes were incubated overnight at 4 °C with primary antibodies against Gal-3, TGF-β1, BNP, and β-actin, followed by an HRP-conjugated secondary antibody (1 h, RT). Bands were detected by ECL (catalog no. 32106; Thermo Fisher Scientific) using a ChemiDoc XRS+ system (Bio-Rad Laboratories) and densitometrically quantified in Fiji ImageJ and normalized to β-actin.

### Immunohistochemistry

IHC was performed on 5-µm paraffin sections following antigen retrieval in 10 mM sodium citrate buffer (pH 6.0, 95–98 °C, 45 min). Endogenous peroxidase was quenched with 3% H_2_O_2_ (15 min) and non-specific binding blocked with 2% normal goat serum (catalog no. S-1000-20; Vector Laboratories) for 60 min. Sections were incubated overnight at 4 °C with primary antibodies against Gal-3, TGF-β1, and BNP, followed by biotinylated secondary antibody, VECTASTAIN^®^ ABC-HRP (catalog no. PK-4000; Vector Laboratories), and AEC substrate (catalog no. SK-4200; Vector Laboratories) (Table 2). Isotype-matched negative controls were processed in parallel. Sections were counterstained with Gill 2 hematoxylin diluted 1:5 and cover slipped. Three non-consecutive sections per animal (3–5 fields/section) were evaluated blindly; mean staining intensity was quantified in Fiji ImageJ.

**Table 2:** Primary and secondary antibodies used for Western blot and IHC

Antibody	Supplier	Catalog	Dilution in WB	Dilution in IHC
**Primary antibodies**				
GAL-3	Proteintech	14979-1-AP	1:1000	1:200
TGF-β1	Proteintech	26155-1-AP	1:2000	1:100
BNP	MyBiosource	MBS2004972	1:1000	1:50
**Secondary antibodies**				
Anti-mouse	Novus biologicals	NB7544	1:3000	–
Anti-rabbit	Thermofisher scientific	A16023	1:2000	–
Goat anti-mouse	Vector Laboratories	BP-2000-50	–	Ready-to-use
Goat anti-rabbit	Vector Laboratories	BP-9100-50	–	Ready-to-use

### Statistics

Statistical analyses were performed using GraphPad Prism 10. Data are presented as mean ± SD. Cardiomyocyte fiber diameter was analyzed by one-way ANOVA followed by Tukey’s post hoc test, as this endpoint was compared across three experimental groups: NML, HYL, and HYG. Grouped molecular datasets, including qRT-PCR, Western blot densitometry, and IHC staining intensity, were analyzed by two-way ANOVA, with experimental condition and molecular marker/protein form as the two factors. Post hoc multiple comparisons were performed within each marker where appropriate. Pearson correlation analysis was used to assess associations between fasting glucose and transcript abundance in the HYG group. Statistical significance was set at *p* < 0.05; ****p < 0.0001.

## Results

### Histological analysis

**Fig. 1:**
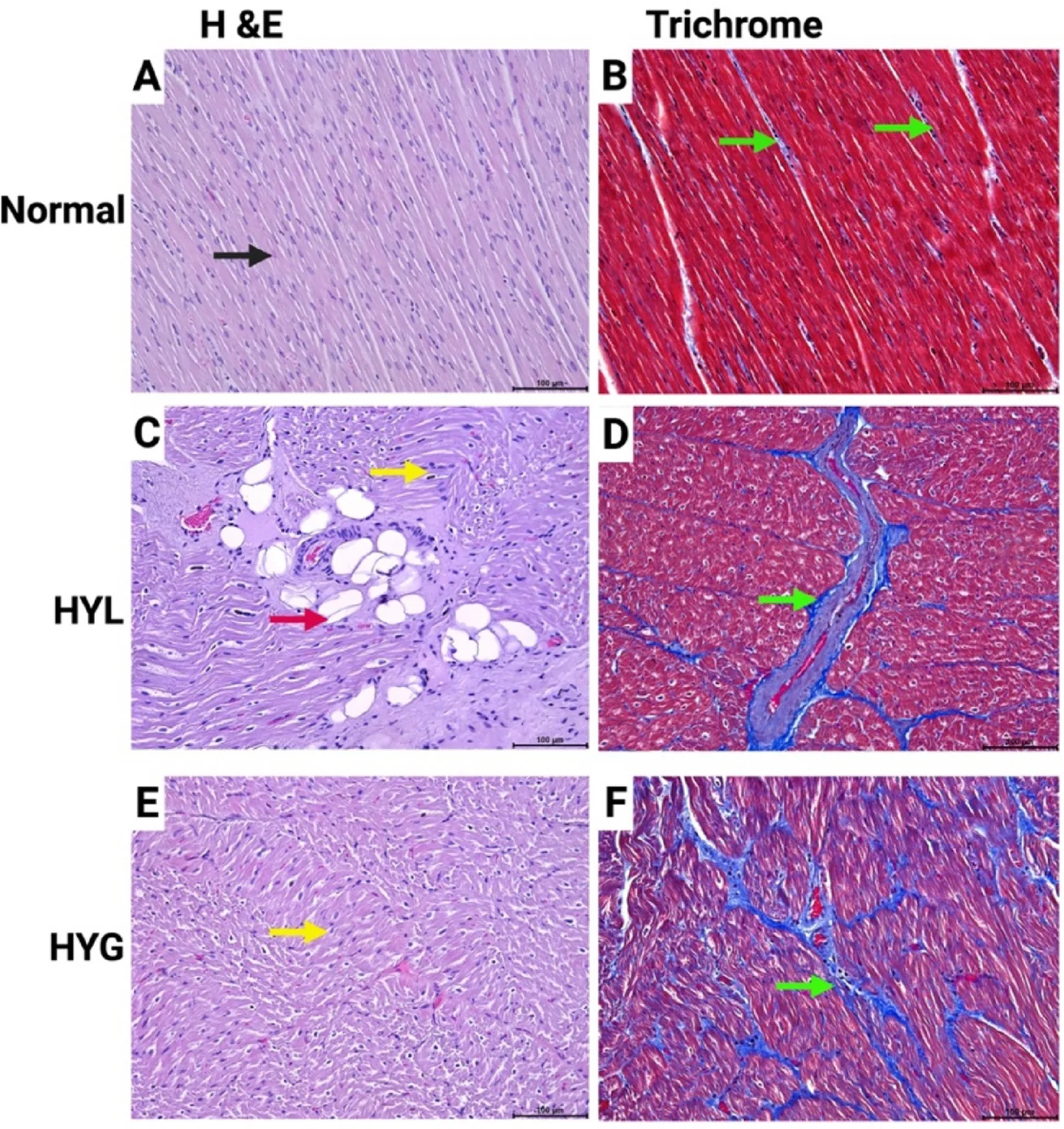
H&E and Masson’s trichrome staining of left ventricular myocardium. Representative sections from normal (**A**, **B**), hyperlipidemic (HYL; **C**, **D**), and hyperglycemic (HYG; **E**, **F**) miniswine. Normal myocardium shows uniform cardiomyocyte alignment and minimal collagen deposition (**A**, **B**). HYL myocardium exhibits focal lipid vacuolation (**C**, red arrow), cardiomyocyte hypertrophy (**C**, yellow arrow), and moderate perivascular fibrosis (**D**, green arrow). HYG myocardium demonstrates marked hypertrophy with wavy fiber disorganization (**E**, yellow arrow) and diffuse interstitial and perivascular collagen deposition (**F**, green arrow). Scale bars = 100 μm; *n* = 6 per group

Hematoxylin and eosin (H&E) staining and Masson’s trichrome staining revealed progressive, group-dependent deterioration of myocardial microarchitecture (Fig. 1). Control myocardium exhibited uniformly aligned cardiomyocytes with centrally positioned nuclei, intact intercalated discs, and no evidence of cellular hypertrophy or inflammatory infiltration (Fig. 1A). Masson’s trichrome confirmed a physiologic extracellular matrix (ECM) scaffold, with fine collagen fibrils restricted to perivascular and endomysial compartments and minimal interstitial deposition (Fig. 1B, green arrows).

In the hyperlipidemic (HYL) group, H&E staining demonstrated focal intracellular lipid vacuolation (Fig. 1C, red arrow) within hypertrophied cardiomyocytes exhibiting disorganized longitudinal fiber alignment (Fig. 1C, yellow arrow). Masson’s trichrome revealed moderate, predominantly perivascular collagen deposition (Fig. 1D, green arrow), consistent with early lipotoxicity-driven fibrotic remodeling of the myocardial interstitium.

Hyperglycemic (HYG) myocardium exhibited a more pronounced pathological phenotype. H&E staining demonstrated marked cardiomyocyte hypertrophy, nuclear enlargement, and loss of uniform fiber parallelism, with a characteristic wavy fiber morphology (Fig. 1E, yellow arrow). Masson’s trichrome revealed extensive, diffuse collagen deposition throughout the interstitial and perivascular compartments (Fig. 1F, green arrow), indicative of an advanced, sustained profibrotic state exceeding that observed in HYL myocardium.

**Fig. 2:**
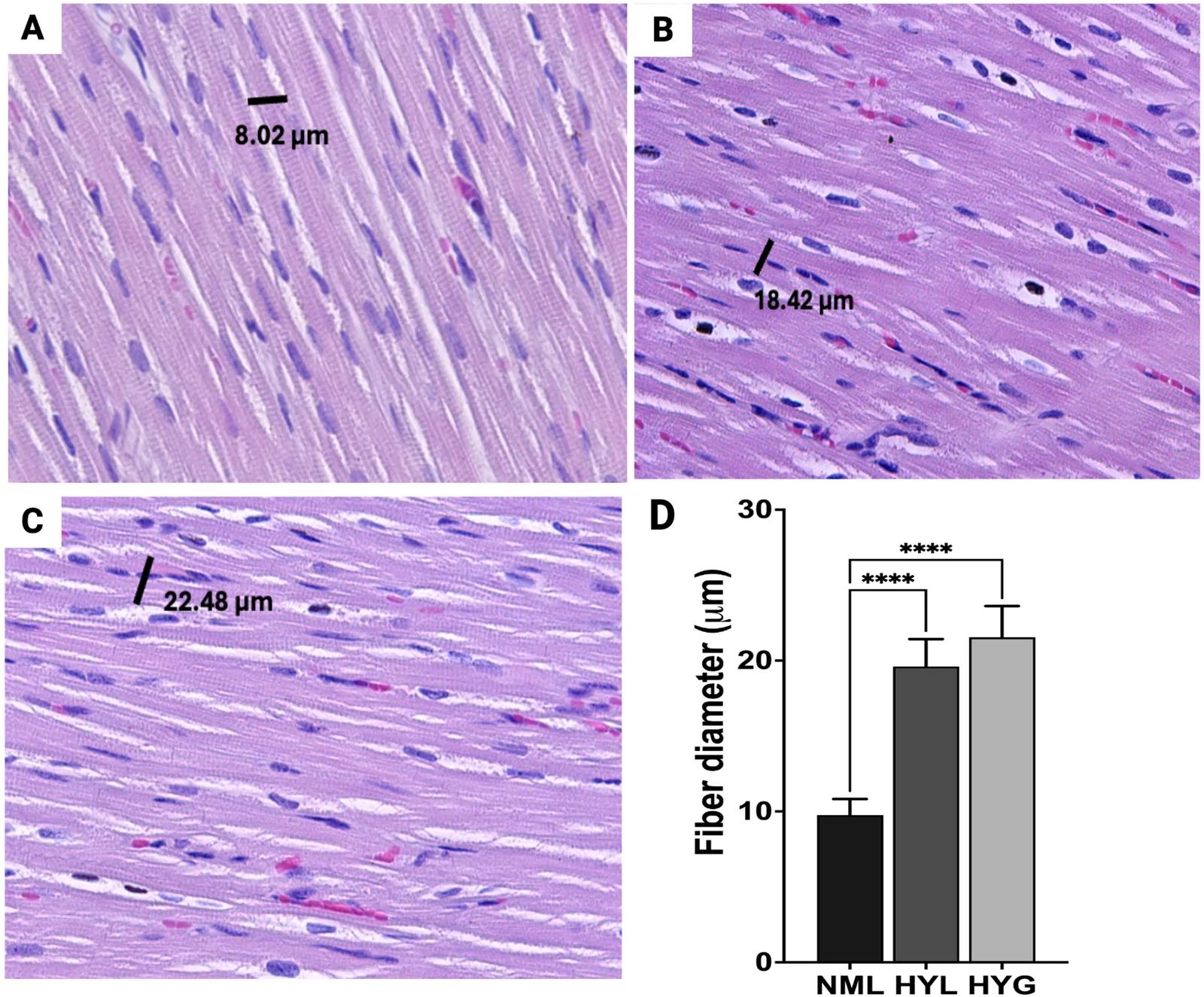
Morphometric quantification of cardiomyocyte fiber diameter in left ventricular myocardium. Representative H&E-stained sections from normal (NML) (**A**), hyperlipidemic (HYL; **B**), and hyperglycemic (HYG; **C**) miniswine. **D** Semi-quantitative bar graph of mean cardiomyocyte fiber diameter across experimental groups. (****p < 0.0001, one-way ANOVA, Tukey’s post-hoc test). Data presented as mean ± SD. Scale bars = 100 μm; *n* = 6 per group

Morphometric quantification of cardiomyocyte fiber diameter, measured from H&E-stained sections as a quantitative index of hypertrophy, confirmed significantly greater fiber diameter in HYL (19.83 μm) and HYG (22.48 μm) myocardium relative to normal controls (9.76 μm; *p* < 0.0001; Fig. 2). HYG myocardium exhibited the greatest fiber diameter across all groups, concordant with the more advanced hypertrophic and fibrotic phenotype observed histologically.

### Gene expression analysis by RT-PCR

**Fig. 3:**
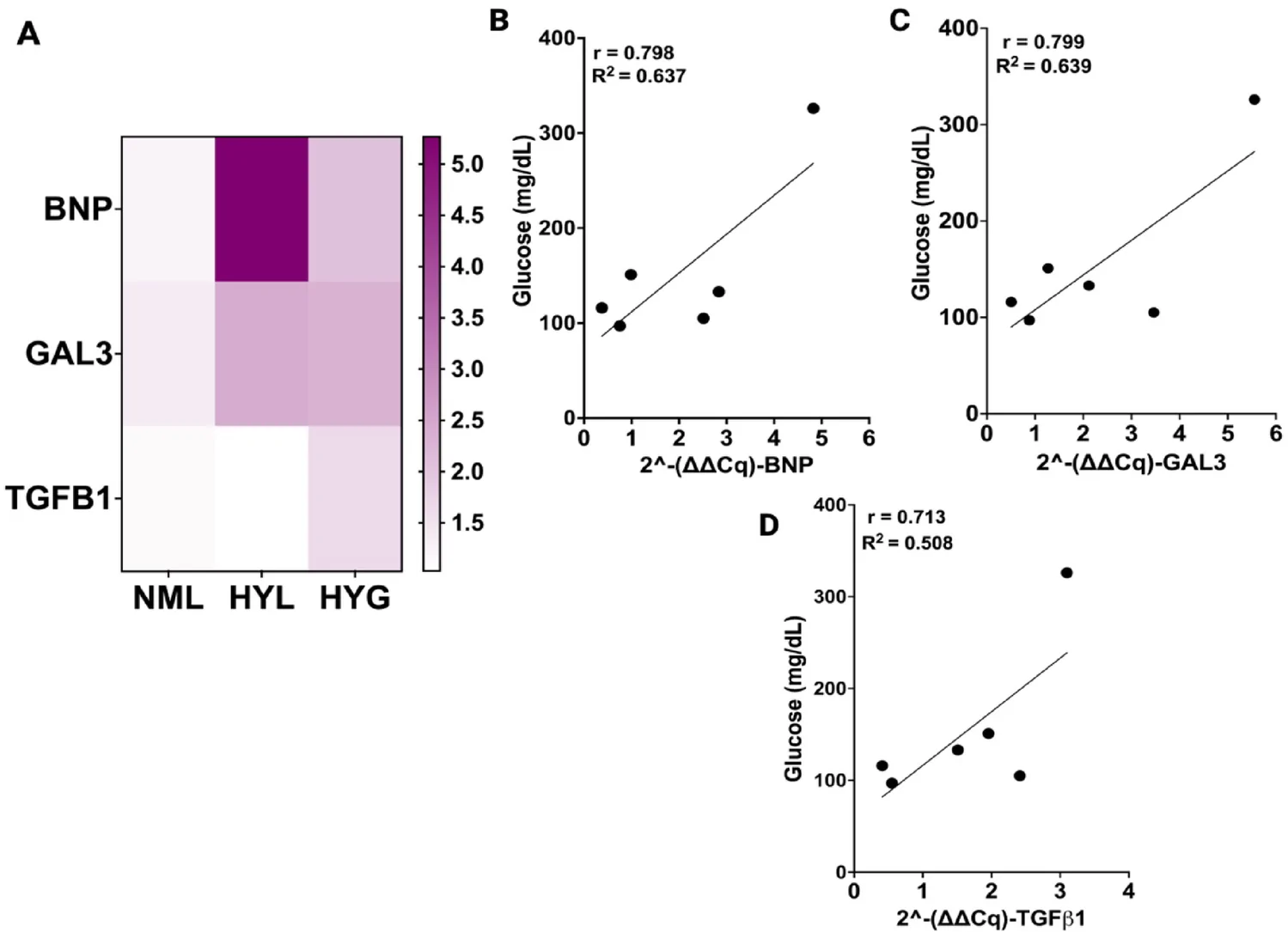
qRT-PCR analysis of myocardial Gal-3, TGF- β1, and BNP expression. **A** Heat map of normalized 2^−(ΔΔCq)^ values across normal, HYL, and HYG groups. **B**–**D** Pearson’s correlation of fasting blood glucose (mg/dL) vs. 2^−(ΔΔCq)^ for BNP (B; *r* = 0.798, R^2^ = 0.637), Gal-3 (C; *r* = 0.799, R^2^ = 0.639), and TGF- β1 (D; *r* = 0.713, R^2^ = 0.508) within the HYG group. Expression normalized to 18S rRNA and expressed as fold-change relative to normal controls by the 2^−ΔΔCT^ method. *n* = 6 per group

Heat map analysis of normalized 2^−(ΔΔCq)^ values showed differential upregulation of all three target transcripts in HYL and HYG myocardium compared with normal controls (Fig. 3A). Gal-3 transcript levels were elevated 2.42-fold in HYL and 2.30-fold in HYG myocardium; TGF-β1 was marginally upregulated in HYL (1.03-fold), but more substantially elevated in HYG myocardium (1.66-fold), indicating that chronic hyperglycemia more potently activates profibrotic transcriptional programs than hyperlipidemia. BNP showed the greatest fold-change, with a 5.27-fold increase in HYL and 2.05-fold in HYG myocardium relative to controls, reflecting a strong natriuretic peptide response to lipid-induced wall stress. Pearson’s correlation analysis within the HYG group further demonstrated significant positive associations between circulating glucose concentration (mg/dL) and myocardial transcript abundance of BNP (*r* = 0.798, R^2^ = 0.637; Fig. 3B), Gal-3 (*r* = 0.799, R^2^ = 0.639; Fig. 3C), and TGF-β1 (*r* = 0.713, R^2^ = 0.508; Fig. 3D), demonstrating a dose-dependent relationship between the degree of hyperglycemia and cardiac remodeling gene induction.

### Western blot analysis

**Fig. 4:**
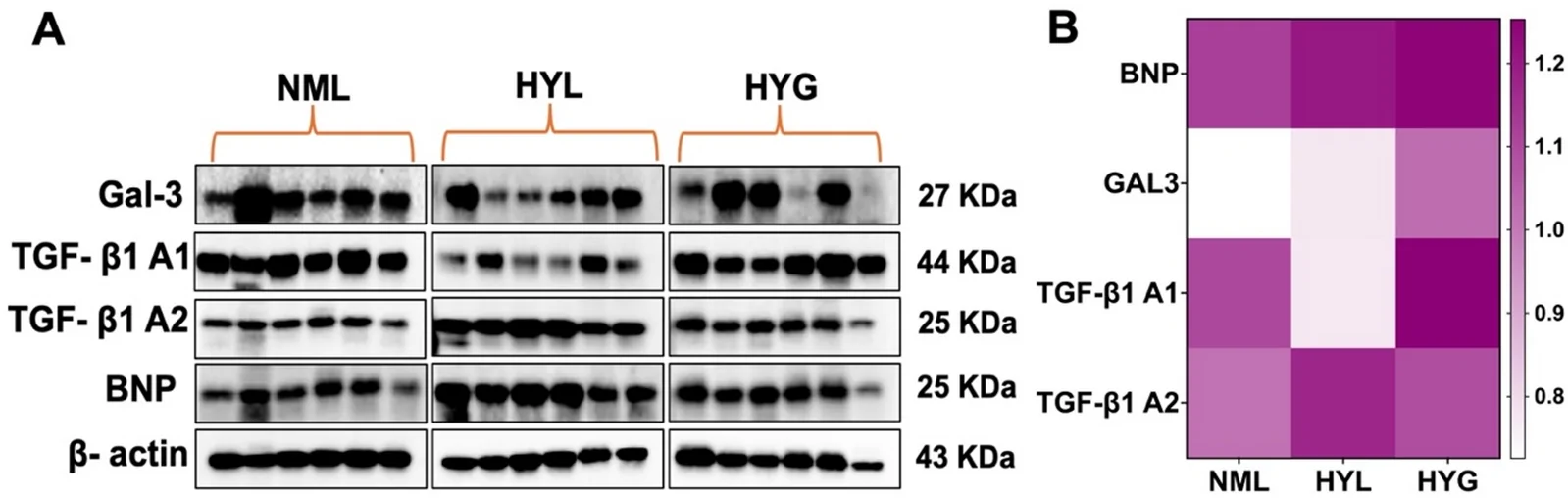
Western blot analysis of Gal-3, TGF-β1, and BNP protein expression. **A** Representative blots for Gal-3, TGF-β1 A1, TGF-β1 A2, and β-actin across normal (NML), HYL, and HYG groups. **B** Densitometric heat map of β-actin-normalized band intensities. *n* = 6 per group

Western blot analysis confirmed condition-dependent alterations in Gal-3, TGF-β1, and BNP protein abundance. All band intensities were normalized to β-actin (43 kDa) as a loading control (Fig. 4A). The densitometric heat map further illustrated group differences in normalized protein expression (Fig. 4B). Gal-3 was resolved at 27 kDa and BNP at 25 kDa. Both proteins showed increased band intensity in HYL and HYG myocardium relative to normal controls (NML), confirmed by heat map densitometry (Fig. 4B). Two immunoreactive bands were resolved for TGF-β1, the latent precursor form (TGF-β1 A1; 44 kDa) and the proteolytically processed, biologically active form (TGF-β1 A2; 25 kDa). TGF-β1 A1 band intensity was greatest in NML and HYG myocardium, whereas TGF-β1 A2 intensity was relatively greater in HYL myocardium (Fig. 4A and B). These findings suggest possible condition-associated differences in TGF-β1 processing or accumulation. In HYL myocardium, the relatively greater lower molecular weight TGF-β1 band may be consistent with increased processing of latent TGF-β1. In contrast, the greater latent precursor signal in HYG myocardium may reflect increased precursor accumulation. However, because downstream SMAD2/3 activation and protease activity were not directly assessed, these findings should be interpreted as associative rather than definitive evidence of distinct TGF-β1 signaling mechanisms.

### Immunohistochemical localization of Gal-3, TGF-β1, and BNP

**Fig. 5:**
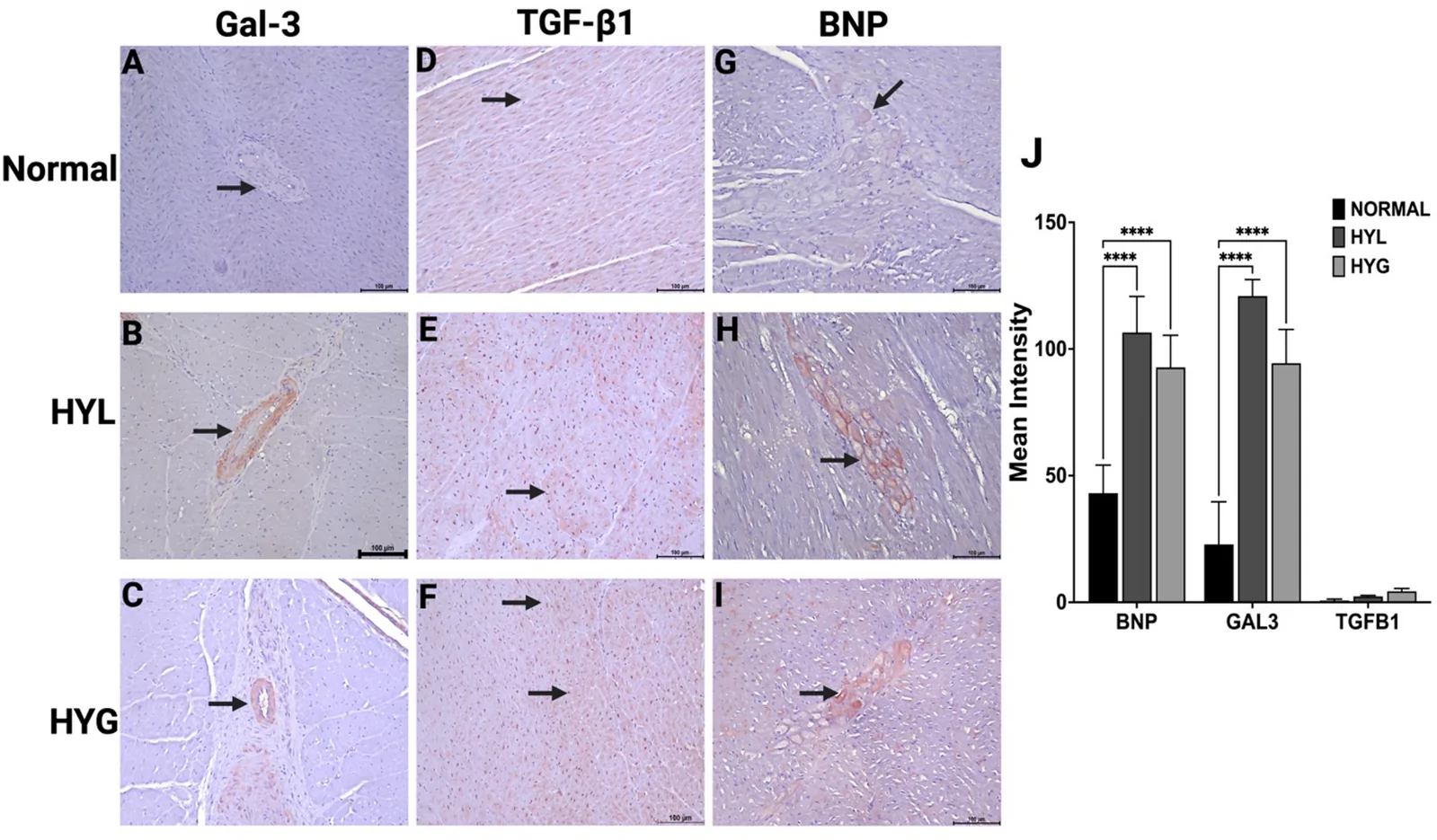
Immunohistochemical localization of Gal-3 (**A**–**C**), TGF-β1 (**D**–**F**), and BNP (**G**–**I**) in LV myocardium with semi-quantitative analysis (**J**). AEC chromogen (red-brown) with hematoxylin counterstain. Gal-3 staining is negligible in normal myocardium (**A**) and markedly increased in the perivascular media in HYL (**B**) and HYG (**C**). TGF-β1 shows low cytoplasmic signal in normal tissue (**D**) with increased cytoplasmic and perinuclear immunoreactivity in HYL (**E**) and HYG (**F**). BNP is absent in normal myocardium (**G**) and strongly localized to Purkinje fibers in HYL (**H**) and HYG (**I**). **J** Mean staining intensity for BNP and Gal-3 was significantly elevated in HYL and HYG vs. normal (****p < 0.0001). TGF-β1 showed a non-significant trend. Data: mean ± SD. Scale bars = 100 μm; *n* = 6 per group

Gal-3 immunoreactivity was negligible in normal myocardium, with only faint AEC signal at a small perivascular focus (Fig. 5A, arrow). In HYL myocardium, pronounced AEC-positive Gal-3 staining was concentrated within the tunica media of intramyocardial vessels (Fig. 5B, arrow), a region morphologically consistent with vascular smooth muscle cell-rich tissue. Similarly, HYG myocardium exhibited positive immunoreactivity within the tunica media of intramyocardial vessels, although overall signal intensity was comparatively diminished relative to the HYL condition. Because cell-type-specific co-localization was not performed, the cellular source of Gal-3 immunoreactivity cannot be definitively assigned. Semi-quantitative analysis of mean AEC staining intensity confirmed a statistically significant elevation of Gal-3 in both HYL and HYG groups relative to normal controls (*p* < 0.0001; Fig. 5J). TGF-β1 immunoreactivity was low and diffusely cytoplasmic in normal cardiomyocytes (Fig. 5D, arrow), with increased cytoplasmic and perinuclear staining observed in both HYL (Fig. 5E, arrow) and HYG (Fig. 5F, arrows) myocardium, consistent with active TGF-β1 synthesis and Golgi-associated pro-peptide processing before secretion. Staining was more diffuse and spatially widespread in HYG tissue (Fig. 5F), concordant with greater transcript upregulation in the HYG group by qRT-PCR; however, mean TGF-β1 staining intensity did not reach statistical significance between groups (Fig. 5J). BNP immunoreactivity was negligible in normal myocardium (Fig. 5G, arrow); in both HYL and HYG conditions, strong focal AEC-positive BNP signal was localized to large, morphologically distinct subendocardial cells consistent with Purkinje fibers, identified by their characteristic large cell diameter, pale cytoplasm, and perinuclear glycogen-rich morphology (Fig. 5H and I, arrows). Mean BNP staining intensity was greatest in the HYL group, followed by HYG, and was lowest in normal myocardium; both experimental groups were significantly elevated relative to normal controls (*p* < 0.0001; Fig. 5J), consistent with early myocardial metabolic dysregulation.

## Discussion

The present study examined metabolic stress-associated cardiac remodeling in a Yucatan miniswine model of HYL and HYG, focusing on Gal-3, TGF-β1, and BNP expression in LV myocardium. Both metabolic conditions were associated with cardiomyocyte hypertrophy, fibrosis, and increased expression of remodeling-related markers. However, the patterns differed between groups. HYL myocardium showed moderate perivascular fibrosis, increased Gal-3 and BNP expression, and relatively greater active TGF-β1 signal. HYG myocardium showed more diffuse collagen deposition, greater TGF-β1 transcript induction, and accumulation of latent TGF-β1 precursor. These findings suggest overlapping but condition-associated remodeling patterns rather than definitive mechanistic separation.

In normal myocardium, a physiologic ECM scaffold composed of fibrillar collagen types I and III provides structural integrity, transmits contractile force, and maintains cardiomyocyte alignment during the cardiac cycle [38]. Disruption of this homeostatic collagen network through pathological activation of cardiac fibroblasts and their differentiation into α-smooth muscle actin-expressing myofibroblasts represents the cellular hallmark of maladaptive cardiac remodeling [39, 40]. In the present study, Masson’s trichrome staining revealed group-dependent differences in collagen deposition that were qualitatively consistent with the molecular findings. HYL myocardium exhibited moderate perivascular and interstitial fibrosis accompanied by intracellular lipid vacuolation, consistent with lipotoxicity-driven ECM remodeling. Elevated circulating free fatty acids in hyperlipidemia promote ectopic myocardial lipid accumulation, generating ceramide, diacylglycerol, and reactive lipid intermediates that activate oxidative stress, macrophage infiltration, and inflammatory cytokine release, thereby driving fibroblast activation independent of ischemic injury [41, 42]. HYG myocardium exhibited a more advanced profibrotic phenotype, with diffuse interstitial and perivascular collagen deposition extending throughout the myocardial parenchyma. Chronic hyperglycemia activates multiple converging profibrotic mechanisms, including AGE-RAGE signaling, mitochondrial ROS generation, NF-κB-mediated inflammatory pathway activation, and direct stimulation of ECM-producing myofibroblasts [31, 43]. The greater severity and spatial extent of collagen deposition in HYG myocardium relative to HYL reflect a more sustained profibrotic state, supported by the differential transcriptional upregulation of TGF-β1 and progressive accumulation of the latent TGF-β1 precursor observed in the hyperglycemic condition. In support of these histological observations, morphometric quantification of cardiomyocyte fiber diameter confirmed significantly greater fiber dimensions in HYL (19.83 μm) and HYG (22.48 μm) myocardium relative to normal controls (9.76 μm; *p* < 0.0001; Fig. 2), providing independent quantitative evidence of pathological cardiomyocyte hypertrophy under both metabolic conditions.

**Fig. 6:**
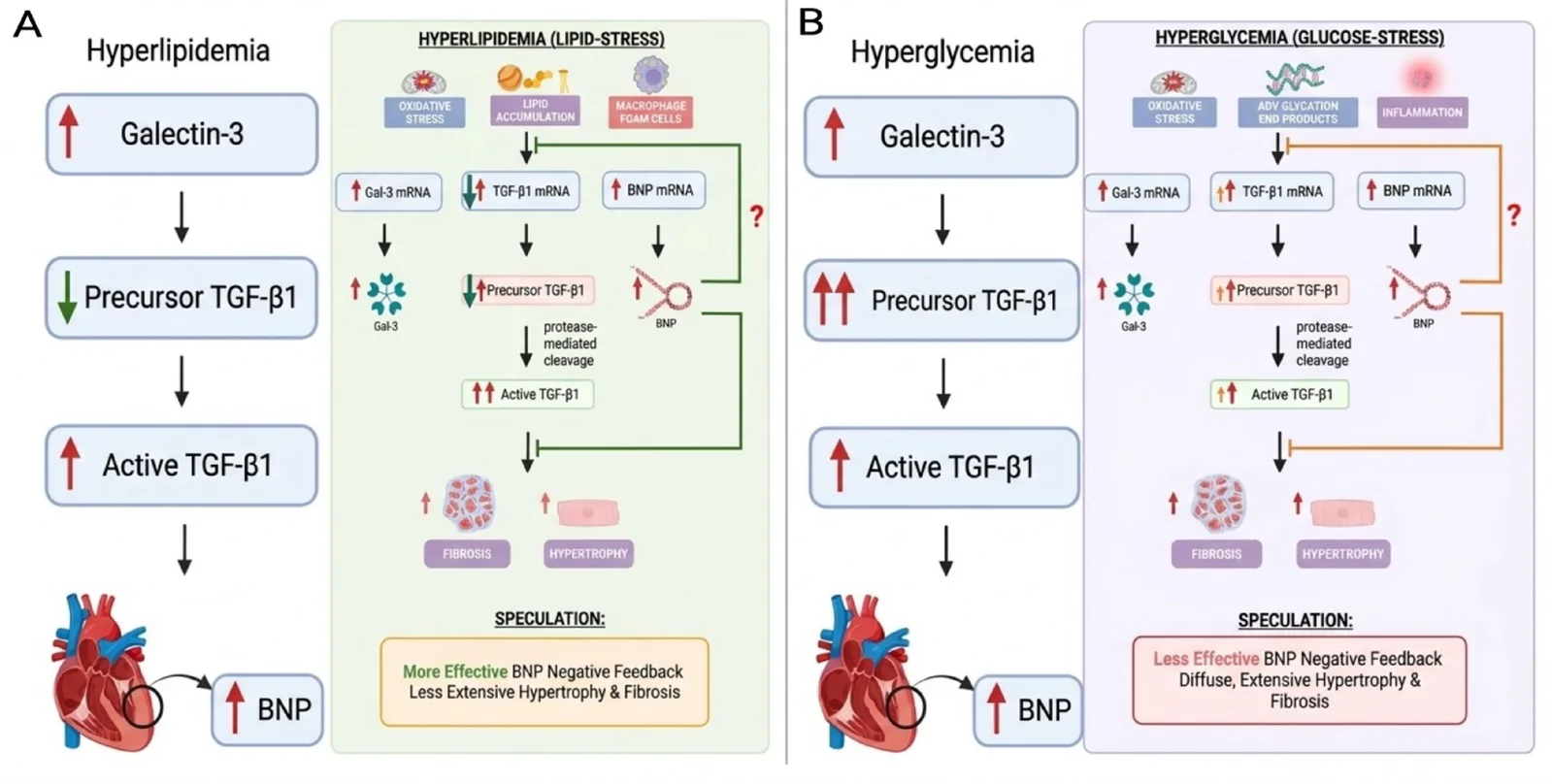
Integrated proposed model of HYL- and HYG-associated cardiac remodeling. **A** Hyperlipidemic stress was associated with increased Gal-3, increased active TGF-β1, reduced precursor TGF-β1, and increased BNP expression, suggesting remodeling with comparatively less extensive fibrosis and hypertrophy. **B** Hyperglycemic stress was associated with increased Gal-3, increased precursor and active TGF-β1, and increased BNP expression, suggesting more diffuse fibrosis and hypertrophy. BNP upregulation may reflect a compensatory stress response; however, BNP signaling efficacy was not directly assessed. This schematic is a proposed, hypothesis-generating model because protease activity, SMAD signaling, BNP receptor/cGMP/PKG signaling, and cell-specific localization were not evaluated

TGF-β1 serves as the central downstream effector of profibrotic signaling in both conditions, yet its mode of dysregulation differed markedly between HYL and HYG myocardium, as illustrated in Fig. 6. TGF-β1 is secreted and stored in the ECM as a biologically inactive large latent complex (LLC) requiring proteolytic cleavage of the latency-associated peptide (LAP) for activation [44, 45]. Once activated, TGF-β1 signals through SMAD2/3 phosphorylation to promote myofibroblast differentiation, upregulate collagen type I and III synthesis, and suppress ECM degradation, collectively producing the fibrosis characteristic of pathological cardiac remodeling [32, 46, 47]. In HYL myocardium, TGF-β1 transcript levels were only minimally elevated (1.03-fold), yet the active TGF-β1 A2 form (~ 25 kDa) predominated on Western blot over the latent A1 precursor (~ 44 kDa). As shown in Fig. 6, oxidative stress, lipid accumulation, and macrophage foam cell-derived proteases in the HYL condition are proposed to drive post-translational proteolytic cleavage of pre-existing latent TGF-β1 in the absence of significant transcriptional induction. ROS and MMP-mediated LAP cleavage are well-established non-transcriptional mechanisms of latent TGF-β1 activation during tissue remodeling [48, 49], explaining how active TGF-β1 signaling and early fibrosis can occur in HYL myocardium despite limited TGF-β1 gene induction. In contrast, in HYG myocardium, TGF-β1 mRNA was substantially more upregulated (1.66-fold), and the latent TGF-β1 A1 precursor predominated, consistent with sustained transcriptional induction and progressive accumulation of a latent precursor reservoir. As depicted in Fig. 6, AGE-RAGE signaling, oxidative stress, and NF-κB-driven inflammation under chronic glucose toxicity converge to sustain persistent TGF-β1 transcription, generating a self-replenishing latent pool for ongoing protease-mediated TGF-β1 activation and SMAD-dependent profibrotic signaling over time [50]. This mechanism is consistent with the more diffuse and extensive collagen deposition observed histologically in HYG myocardium, as well as prior reports linking TGF-β1-driven transcription of collagen types I and III to interstitial fibrosis in diabetic cardiomyopathy.

As illustrated in both Fig. 6A and B, Gal-3 appeared to be a shared remodeling-associated marker across both metabolic conditions. Gal-3, a β-galactoside-binding lectin secreted predominantly by classically activated (M1) macrophages, promotes cardiac fibroblast activation and amplifies TGF-β1-dependent ECM remodeling through β1-integrin receptor binding and downstream FAK/PI3K/Akt/SMAD2/3 signaling [51, 52]. This Gal-3/TGF-β1 signaling axis has been mechanistically validated in atrial fibrillation, where Gal-3 overexpression directly activated TGF-β1/Smad2/3 signaling and promoted structural atrial remodeling [35, 53]. In the present study, Gal-3 transcript levels were increased in both HYL and HYG myocardium, and IHC showed perivascular Gal-3 enrichment. However, because macrophage, fibroblast, and vascular smooth muscle cell co-localization was not performed, the cellular source of Gal-3 cannot be definitively assigned. These findings support involvement of a perivascular inflammatory-fibrotic response but require future cell-specific validation.

BNP is commonly induced in response to myocardial stretch and wall stress and may exert compensatory natriuretic, vasodilatory, and antifibrotic effects through natriuretic peptide receptor/cGMP/PKG signaling [54, 55]. Natriuretic peptide signaling has also been reported to oppose TGF-β-dependent fibroblast activation and collagen synthesis [26, 56]. In the present study, BNP transcript abundance increased in both metabolic groups, with a greater fold-change in HYL than in HYG myocardium. BNP immunoreactivity was predominantly localized to morphologically distinct subendocardial Purkinje fibers rather than diffusely distributed throughout the working myocardium. Therefore, the BNP findings should be interpreted as evidence of a stress-associated response rather than direct proof of effective global antifibrotic signaling. Although BNP expression was increased in HYG myocardium, this increase may not necessarily translate into functional antifibrotic protection. Future studies measuring circulating BNP levels, BNP receptor expression, cGMP/PKG activation, and cell-specific BNP localization are needed to determine whether BNP signaling provides effective counter-regulatory protection under metabolic stress.

The molecular framework established in the present study, and graphically summarized in Fig. 6, identifies several therapeutically tractable targets for metabolic cardiomyopathy. Pharmacological inhibition of Gal-3 using modified citrus pectin or the inhaled galectin-3 inhibitor TD139, and enhancement of natriuretic peptide signaling through neprilysin inhibition with sacubitril/valsartan, have demonstrated antifibrotic efficacy in preclinical and clinical settings [57–59]. These strategies demand evaluation in models of combined lipid and glucose metabolic injury to determine their capacity to interrupt the Gal-3/TGF-β1 fibrogenic axis and restore BNP counter-regulatory efficacy in the setting of chronic cardiometabolic stress.

Together, these findings indicate that hyperlipidemic and hyperglycemic metabolic stress promote myocardial remodeling through overlapping yet distinct molecular mechanisms. Gal-3 likely functions as a shared upstream mediator of the inflammatory-fibrotic signaling pathway, while TGF-β1 acts as a central downstream effector driving ECM deposition. BNP, however, likely represents a compensatory response that counteracts myocardial stress and limits fibrosis. The differential regulation of these pathways under chronic lipid and glucose metabolic stress may contribute to the distinct structural remodeling patterns observed histologically.

From a translational perspective, the coordinated changes in Gal-3, TGF-β1, and BNP may have relevance for biomarker development and therapeutic stratification in metabolic cardiomyopathy. Gal-3 may reflect inflammatory-fibrotic remodeling, TGF-β1 may indicate activation of profibrotic extracellular matrix signaling, and BNP may represent myocardial stress and compensatory natriuretic peptide activation. The differential expression patterns observed under lipid- and glucose-dominant stress suggest that combined biomarker assessment may provide more information than any single marker alone. Therapeutically, the Gal-3/TGF-β1 axis may represent a potential antifibrotic target, whereas strategies that preserve or enhance natriuretic peptide signaling may help limit maladaptive remodeling. However, these translational implications remain exploratory and require validation in longitudinal studies, combined HYL + HYG models, and studies that include functional cardiac outcomes.

## Limitations and future directions

Certain limitations of the present study highlight important directions for future investigation. Tissue collection at a single experimental time point limits characterization of the progressive expression of Gal-3, TGF-β1, and BNP during cardiac remodeling. Longitudinal sampling at defined intervals would delineate the sequential activation of profibrotic and counter-regulatory signaling cascades under HYL and HYG conditions. Downstream TGF-β1 signaling was not directly assessed in the present study. Future studies should quantify SMAD2/3 phosphorylation, α-SMA, collagen I/III expression, MMP-2/9 activity, and ROS generation to determine whether the observed TGF-β1 banding patterns reflect differential pathway activation. BNP signaling was also not functionally evaluated, as circulating BNP levels, BNP receptor expression, cGMP/PKG activation, and cell-specific BNP localization were not assessed. Therefore, conclusions regarding BNP counter-regulatory efficacy remain inferential. Co-immunostaining for macrophage, fibroblast, vascular smooth muscle cell, and Purkinje fiber markers would help define the cellular sources of Gal-3, TGF-β1, and BNP immunoreactivity. Finally, the absence of a combined HYL + HYG experimental group limits extrapolation to the clinical co-morbidity of hyperlipidemia and type 2 diabetes mellitus; incorporation of this group in future investigations would permit examination of synergistic cardiometabolic mechanisms and their cumulative impact on the Gal-3/TGF-β1/BNP signaling axis.

## Conclusion

This study examined how chronic metabolic conditions like hyperlipidemia and hyperglycemia influenced hypertrophic and fibrotic remodeling of left ventricular myocardium. Both conditions activated molecular markers associated with cardiac remodeling, including Gal-3, TGF-β1, and BNP; however, the patterns of signaling differed between metabolic conditions. Histological analysis demonstrated cardiomyocyte hypertrophy and ECM remodeling in both experimental groups, with more diffuse collagen deposition observed in hyperglycemic myocardium compared to the moderate interstitial and perivascular fibrosis seen in hyperlipidemia. HYG was associated with increased TGF-β1 transcription and sustained profibrotic signaling, further supporting the histological findings. Conversely, hyperlipidemic myocardium demonstrated greater activation of latent TGF-β1 and increased transcription of the BNP gene, consistent with early remodeling and compensatory regulation. These results highlight the complex interplay between inflammatory, profibrotic, and counter-regulatory pathways in metabolic stress and cardiovascular remodeling. Understanding the differential regulation of cardiac remodeling in high-lipid and high-glucose states may provide insight into potential targets for therapeutic interventions.

## Data Availability

All original raw and analyzed data are deposited in the Cloud provided by WesternU and are also available with the corresponding author upon request through the proper channel.
